# Low Psoas-Muscle index is associated with decreased survival in hepatocellular carcinoma treated with transarterial chemoembolization

**DOI:** 10.1080/07853890.2022.2081872

**Published:** 2022-05-31

**Authors:** Jin-Xing Zhang, Hai-Tao Yan, Ye Ding, Jin Liu, Sheng Liu, Qing-Quan Zu, Hai-Bin Shi

**Affiliations:** a Department of Interventional Radiology, The First Affiliated Hospital with Nanjing Medical University, Nanjing, China; b Department of Maternal, Child and Adolescent Health, School of Public Health, Nanjing Medical University, Nanjing, China; c Department of Clinical Medicine Research Institution, The First Affiliated Hospital with Nanjing Medical University, Nanjing, China

**Keywords:** Carcinoma, hepatocellular, chemoembolization, therapeutic, sarcopenia, survival analysis

## Abstract

**Purpose:**

Skeletal muscle index (SMI) is a promising predictor of clinical outcomes in patients with malignant diseases. As a simpler surrogate of sarcopenia-psoas muscle index (PMI), its predict value for overall survival (OS) after transarterial chemoembolization (TACE) for hepatocellular carcinoma (HCC) has not been reported. To determine if changes in the PMI predicted OS in individuals with HCC treated with TACE.

**Patients and Methods:**

A retrospective analysis was performed in HCC patients treated with TACE between January 2018 and March 2019. The survival curve according to PMI was estimated by the Kaplan–Meier method and then compared by the log-rank test. Cox proportional hazards models were conducted to identify the prognostic factors for OS. Furthermore, the predictive abilities of PMI and SMI were compared by using Harrell’s concordance index (C-index).

**Results:**

Two hundred and twenty-eight patients (175 men, mean age 59 ± 11 years) were analysed. The OS was less in patients with low PMI than those with high PMI (median OS: 16.9 vs. 38.5 months, *p* < .001). Multivariate analysis found that either PMI (hazard ratio [HR] = 0.64; 95% confidence interval [CI], 0.45–0.91; *p* < .001) or SMI (HR = 0.51; 95% CI, 0.36–0.72; *p* < .001) was significantly associated with OS. In the multivariate analysis, the C-index for PMI was 0.78 and 0.79 for SMI (*p* = .985).

**Conclusion:**

PMI is a simple tool to predict OS in HCC patients treated with TACE. The predictive ability of PMI is comparable to that of SMI.

**Key messages**
1. Low psoas-muscle index is associated with decreased overall survival in hepatocellular carcinoma treated with transarterial chemoembolization (TACE).
2. Psoas-muscle index has advantages of being faster and easier to acquire, which thus makes it more likely to achieve widespread clinical application.

## Introduction

For patients with unresectable hepatocellular carcinoma (HCC), transarterial chemoembolization (TACE) is a widely accepted treatment [1,2]. Overall survival (OS) following TACE is primarily depended on tumour stage, liver function, and performance status [2–4]. However, protein energy malnutrition is a frequent complication of HCC, often neglected. Routine laboratory indices are limited to evaluate the nutritional status because of their accuracy and variability. Recently, sarcopenia as a metric of performance and nutritional status has attracted attention owing to its possible predictive value [5,6].

Computed tomography (CT) is a well-established tool for diagnosing sarcopenia [7]. For this, skeletal muscle index (SMI) is used and is calculated from the cross-sectional area of major muscle groups at the third lumbar vertebrae (L3) [8,9]. Despite its good performance in prediction, SMI is mostly limited to research and rarely used in clinical decision-making. This may be due, in part, to the specialised radiologic software and expertise needed to determine SMI [7]. Therefore, there is a need to find a simpler index that quantifies sarcopenia.

A single-muscle approach to be used clinically for the diagnosis of sarcopenia is a recent trend in the literature on CT-defined muscle quantification [10] Currently, the psoas muscle is one of the most common muscles for the diagnosis of sarcopenia [11]. However, whether psoas muscle also predict survival is not known. The aim of this retrospective study was to explore whether psoas muscle index (PMI), as defined by the length and width of the psoas muscle, was useful for predicting the OS in HCC patients treated with TACE, and to compare the predictive ability of PMI and SMI for these patients.

## Material and methods

The authors are accountable for all aspects of the work in ensuring that questions related to the accuracy or integrity of any part of the work are appropriately investigated and resolved. The study was conducted in accordance with the Declaration of Helsinki (as revised in 2013). The study was approved by institutional ethics committee of the institution and individual consent for this retrospective analysis was waived. The authors confirm that the data supporting the findings of this study are available within the article and its supplementary materials.

### Patients

In this study, the clinical records of 285 patients with HCC who were initially treated with TACE at our institution were retrospectively reviewed between January 2018 and March 2019. The inclusion criteria were: (1) pathologically or radiologically confirmed HCC; (2) unresectable disease or intolerable resection; (3) Barcelona Clinic Liver Cancer (BCLC) stage A/B/C; and (4) availability of CT imaging. The exclusion criteria were: (1) Child–Pugh class C; (2) Eastern Cooperative Oncology Group (ECOG) performance status > 2; (3) patients who did not have a complete abdominal CT examination covering the level of L3; (4) spontaneous ruptured HCC; (5) patients received liver transplantation or surgical resection following initial TACE; and (6) missing data. Ultimately, we enrolled 228 patients in this study. Clinical data collected included age, gender, weight, height, aetiology, Child–Pugh class, α-Fetoprotein (AFP) level, portal vein thrombus (PVTT), BCLC stage, ALBI [12], SMI, PMI, image findings and clinical outcomes.

### Evaluation of skeletal muscle mass

**Figure 1.**
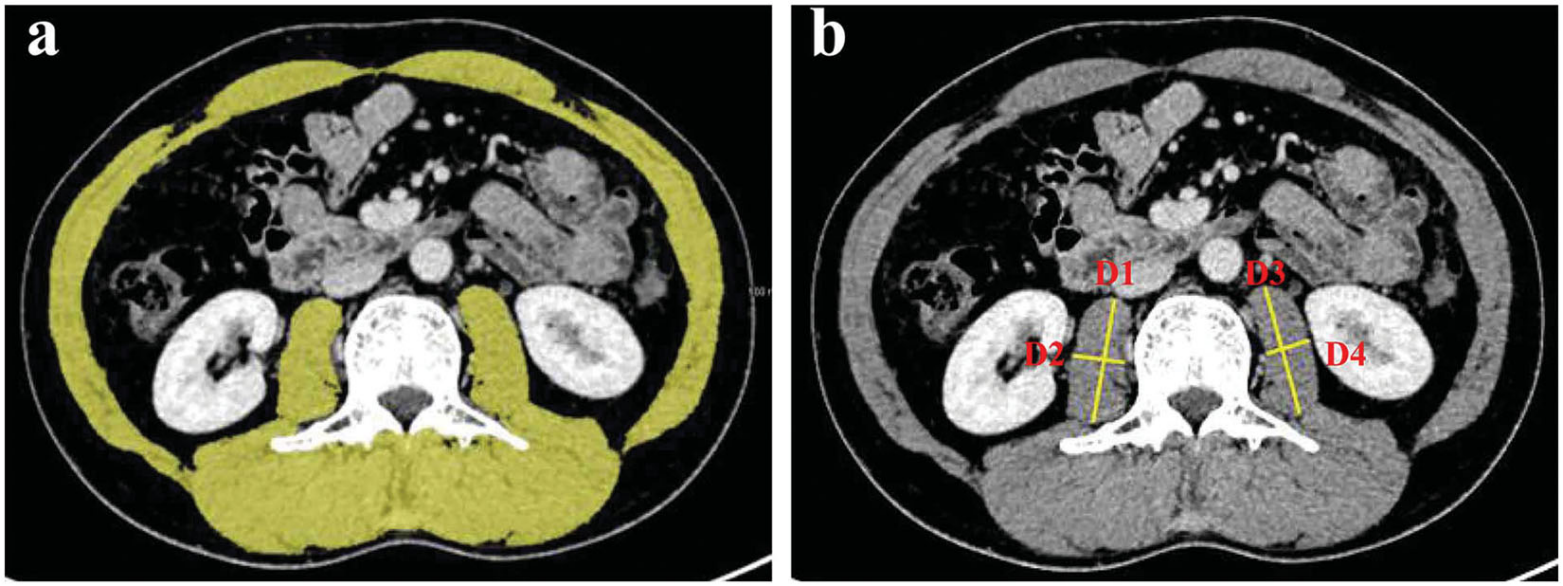
(**a**) Area of skeletal muscle mass in the L3 region defined by outlining the borders of skeletal muscle. (**b**) Measurements of axial and transverse diameters (mm) of bilateral psoas muscle at L3.

Skeletal muscle mass was assessed using CT images acquired for the purpose of HCC evaluation. For the analysis of SMI, venous-phase axial images of the abdomen were exported to a dedicated post-processing workstation (MAGNETOM Skyra, Siemens Healthcare, Germany), which enables tissue demarcation using Hounsfield unit (HU) thresholds. The muscles were quantified within a HU range of −29 to 150 HU [13] and tissue boundaries were manually corrected as needed. The muscles of the L3 region are the psoas, erector spinae, quadratus lumborum, transversus abdominis, external and internal obliques, and rectus abdominis. Normalised the cross-sectional areas of muscle (cm^2^) by the square of the height (m^2^) was done to obtain the SMI (cm^2^/m^2^) [8,14]. A representative image is shown in Figure 1. For PMI, the axial and transverse diameters (mm) of bilateral psoas muscle were measured at the level of mid-L3. Two trained observers blinded to patient details and clinical outcomes individually measured the axial and transverse diameters of bilateral psoas muscle. Afterwards, measurements were averaged between the observers. SMI and PMI were calculated as:

$$\mathrm{SMI}\;({\mathrm{cm}}^{2}/m^{2})=\frac{\text{total muscle area }[{\mathrm{cm}}^{2}]}{\mathrm{height}[m]\times \mathrm{height}[m]}$$

$$\mathrm{PMI}\;(\mathrm{mm}/m^{2})=\frac{\left(D1[\mathrm{mm}]+D2[\mathrm{mm}]+D3[\mathrm{mm}]+D4[\mathrm{mm}]\right)}{\mathrm{height}[m]\times \mathrm{height}[m]}$$

### Therapeutic strategy of TACE

TACE was performed *via* the right femoral artery under local anaesthesia. A 5-F catheter was introduced, and angiography was conducted to assess the tumour number, size, localization, and tumour-feeding arteries. Embolization *via* a microcatheter was performed with lipiodol-based injectant.

### Follow-up

Patients were followed every 1–3 months until the patient died or until the end of the study. Tumour markers, imaging examinations, and liver function were tested. When residual viable tumours were identified or new lesions identified, the patients were treated according to their tumour status and general condition after multidisciplinary discussion. The OS time was defined as the period from initial TACE to death or 31 August 2021.

### Statistical analysis

Continuous data are presented as mean and standard deviation if normally distributed and median (range) if the distribution was skewed for continuous variables. Categorical data are presented as frequency and percentage. Interobserver agreement of the measurements of PMI was assessed using the intraclass correlation coefficient (ICC) and its 95% confidence interval (CI). ICC results were interpreted according to the following criteria: poor (0 to 0.5), moderate (0.50 to 0.75), substantial (0.75 to 0.9), and excellent (0.9 to 1) [15]. Correlation between PMI and SMI was assessed with the Pearson correlation coefficient (*r*). Pearson’s *r* indicates linear correlation between PMI and SMI.

To identify the cut-off points for PMI and SMI that would best predict the OS, maximally selected rank statistics were used [16,17]. Patients were classified into high and low PMI (SMI) groups according to the cut-off value, and estimated OS using the Kaplan–Meier method, and differences between curves using the log-rank test.

Univariate and multivariate Cox’s proportional hazards models were used to evaluate the predictive factors of OS. Factors with *p* < .2 in the univariate analysis were considered potential predictors of OS and were further assessed by multivariate analysis. The predictive abilities of PMI and SMI were evaluated by using Harrell’s concordance index (C-index) [18]. Statistical analysis was performed using R software (version 3.5.1). A *p* value of less than .05 was considered statistically significant.

## Results

### Patient characteristics

A total of 773 TACE procedures were identified, with an average of 3.2 sessions per patient (range, 1–13 sessions). The mean age was 58.9 years, and 76.8% of patients were male. The majority cause of liver disease was hepatitis B virus infection. Two hundred and one (88.2%) patients had Child-Pugh class A liver function. The BCLC stage distribution was as follows: stage A, 58 (25.4%); stage B, 112 (49.1%); and stage C, 58 (25.4%). Among the 58 patients with BCLC stage C, metastasis occurred in 30 cases. The details of patient characteristics are summarized in Table 1.

**Table 1.** Patients characteristics.

Variables	*n* = 228
Age (years)	58.9 ± 11.0
Gender (male/female)	175/53
BMI (M 24 kg/m^2^)	105
Aetiology (HBV/others)	194/34
AFP (> 400 ng/mL)	78
Child-Pugh class (A/B)	201/27
ALBI grade (1/2/3)	104/116/8
Number of tumours (> 3)	63
Maximum tumour diameter (> 3 cm)	144
Portal vein thrombus	40
Metastasis	30
BCLC stage (A/B/C)	58/112/58
SMI (cm^2^/m^2^)	45.3 (27.5-69.4)
PMI (mm/m^2^)	41.8 (26.9-55.5)

Note: BMI: body mass index; AFP: α-Fetoprotein; BCLC: Barcelona Clinic Liver Cancer; SMI: skeletal muscle index; PMI: psoas muscle index.

### Cut-off values for PMI and SMI

**Figure 2.**
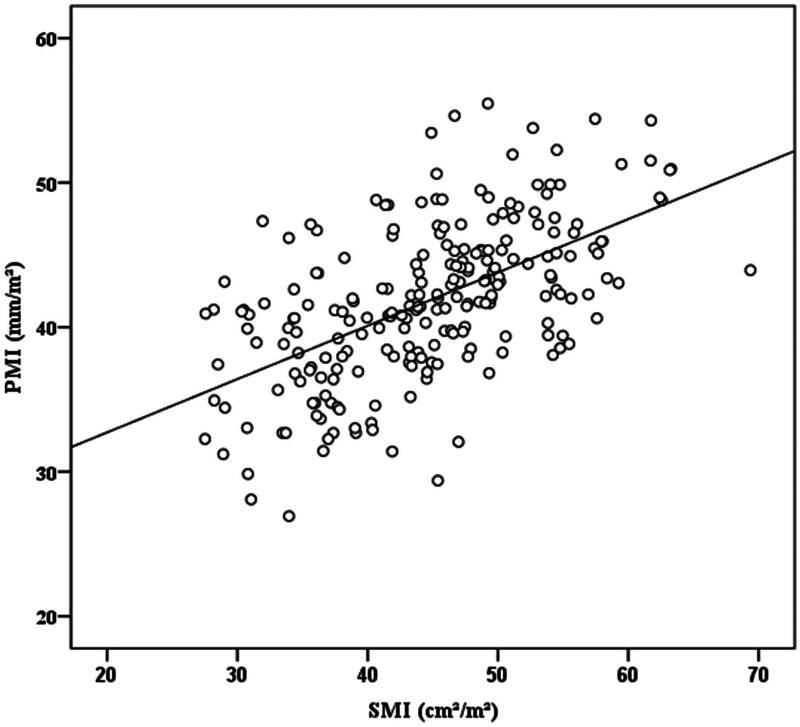
Scatter plot of correlation values between PMI and SMI (*r* = 0.57, *p* < .001).

Interobserver agreement of PMI measurements was excellent with an ICC of 0.96 (95% CI, 0.95–0.97). There was a significant linear (*p* < .001) and moderately strong relationship (*r* = 0.57) between PMI and SMI (Figure 2). As expected, the PMI was significantly greater in men (median: 42.92 mm/m^2^, range: 28.08–55.47) than that in women (median: 39.52 mm/m^2^, range: 26.92 − 53.78; *p* < .001). Given this, gender-specific cut-off values were established by using maximally selected rank statistics. The cut-off values for PMI in men and women were 42.28 and 37.42 mm/m^2^, respectively. Likewise, the cut-off values for SMI in men and women were 45.95 and 33.96 cm^2^/m^2^, respectively.

Supplemental Table 1 shows the patient characteristics classified by PMI. Higher BMI (≥ 24 kg/m^2^), lower ALBI grade [1], and BCLC stage (A) were more frequently in patients with high PMI. While higher AFP level (> 400 ng/mL), large maximum tumour diameter (> 3 cm), and portal vein thrombus were more frequently in patients with low PMI.

Supplemental Table 2 shows the patient characteristics classified by SMI. Higher BMI (≥ 24 kg/m^2^), and BCLC stage (A) were more frequently in patients with high SMI. While older age, female, and large maximum tumour diameter (> 3 cm) were more frequently in patients with low SMI.

### Overall survival

Patients were followed up for a median of 22.3 months. As the last follow-up visit, 134 of the 228 patients (58.7%) had died. The median survival time was 24.2 months (range: 1–41.3 months).

**Figure 3.**
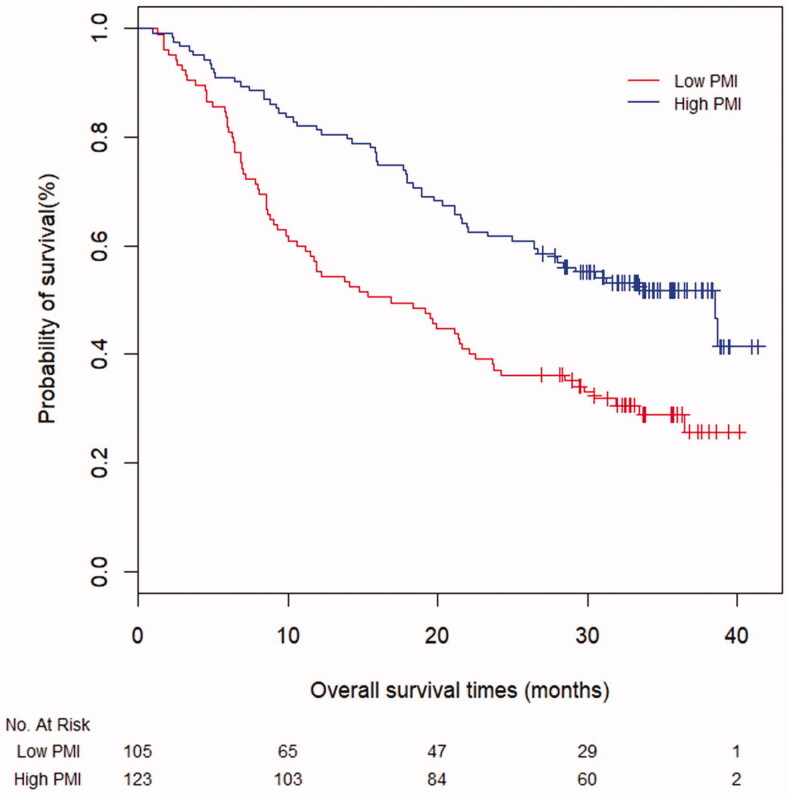
Kaplan–Meier curves for survival time in all patients based on PMI (*p* < .001).

**Figure 4.**
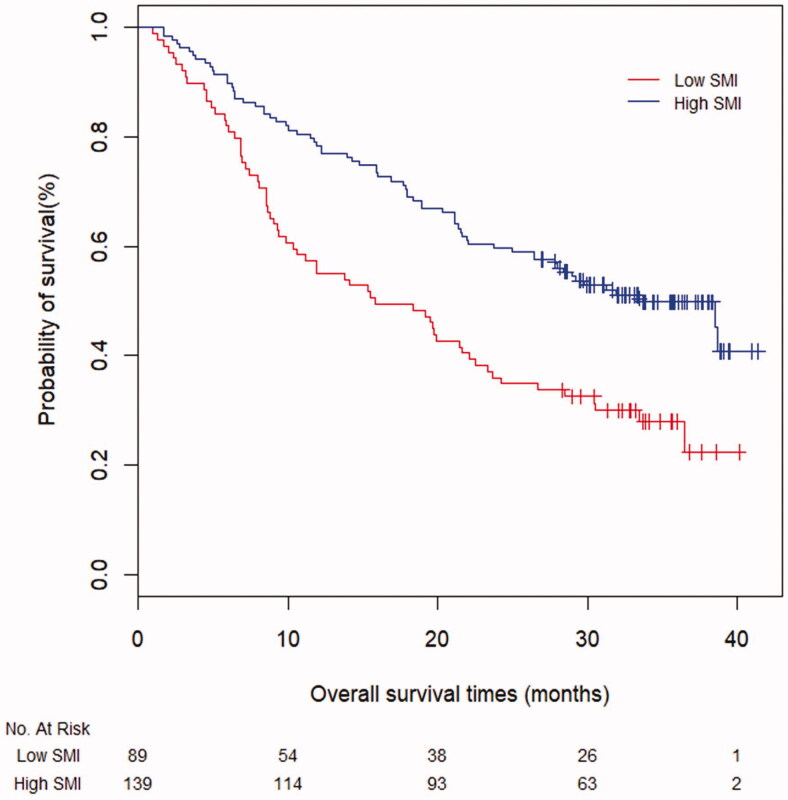
Kaplan–Meier curves for survival time in all patients based on SMI (*p* < .001).

Survival curves for patients classified by cut-off values of PMI and SMI are shown in Figures 3 and 4, respectively. OS were significantly different in patients stratified by PMI (median OS: 16.9 vs. 38.5 months, *p* < .001) and SMI (median OS: 15.8 vs. 33.5 months, *p* < .001).

### Predictive factors for overall survival

On univariate analysis, PMI, SMI, AFP level, Child-Pugh class, ALBI grade, number of tumours, maximum tumour diameter, portal vein thrombus and metastasis were all found to be associated with OS (Table 2). The independent predictive values of PMI and SMI using two multivariate Cox’s proportional hazards models (PMI-Model and SMI-Model) found that both PMI (HR = 0.64; 95% CI, 0.45–0.91; *p* = .014) and SMI (HR = 0.51; 95% CI, 0.36–0.72; *p* < .001) were significant independent prognostic factors associated with OS (Table 2). The predictive ability of the PMI was evaluated in comparison with SMI by means of Harrell’s concordance index. The C-index was 0.78 (95% CI: 0.74–0.81) for PMI and 0.79 (95% CI: 0.76–0.82) for SMI (*p* = .985).

**Table 2.** Univariate and multivariate analysis for predictive factors of overall survival.

	Univariate		Multivariate (PMI-Model)		Multivariate (SMI-Model)	
	HR (95%CI)	*p* Value	HR (95%CI)	*p* Value	HR (95%CI)	*p* Value
Age	0.84 (0.55–1.27)	.410				
Gender (male)	1.00 (0.98–1.01)	.964				
BMI (≥ 24 kg/m^2^)	0.96 (0.68–1.35)	.816				
Aetiology (HBV)	1.18 (0.74–1.86)	.487				
AFP (> 400 ng/mL)	3.86 (2.73–5.45)	<.001	2.46 (1.68–3.60)	<.001	2.81 (1.93–4.10)	<.001
Child-Pugh class (A)	2.26 (1.43–3.58)	.001	2.13 (1.34–3.41)	.002	2.08 (1.30–3.32)	.002
ALBI						
1	1.00 (reference)					
2	1.43 (1.00–2.04)	.047				
3	4.28 (1.92–9.52)	<.001				
Number of tumours (> 3)	3.21 (2.26–4.55)	<.001				
Maximum tumour diameter (>3 cm)	2.43 (1.65–3.57)	<.001	2.01 (1.25–3.24)	.004	2.06 (1.29–3.29)	.002
Portal vein thrombus	3.97 (2.68–5.86)	<.001				
Metastasis	7.65 (4.87–12.02)	<.001	1.82 (1.05–3.16)	.032	1.99 (1.16–3.43)	.013
BCLC stage						
A	1.00 (reference)		1.00 (reference)		1.00 (reference)	
B	2.17 (1.28–3.68)	.004	1.66 (0.97–2.87)	.067	1.60 (0.93–2.75)	.091
C	10.82 (6.25–18.74)	<.001	5.85 (3.06–11.17)	<.001	5.55 (2.90–10.59)	<.001
SMI (low)	0.52 (0.37–0.73)	<.001			0.51 (0.36–0.72)	<.001
PMI (low)	0.42 (0.28–0.63)	<.001	0.64 (0.45–0.91)	.014		

HR: hazard ratio; CI: confidence interval; BMI: body mass index; AFP: α-Fetoprotein; BCLC: Barcelona Clinic Liver Cancer; SMI: skeletal muscle index; PMI: psoas muscle index.

PMI-Model: Adjusted for AFP, Child-Pugh class, maximum tumour diameter, metastasis and BCLC stage; SMI-Model: Adjusted for AFP, Child-Pugh class, maximum tumour diameter, metastasis and BCLC stage.

## Discussion

In this present study, the prognostic role of PMI and SMI in HCC patients undergoing TACE was interrogated. We found PMI and SMI reliable in predicting OS. As well, owing to its simplicity and accessibility, PMI was more convenient for clinical use.

Nutritional status assessment is valuable for patients with HCC, especially in the advanced stages of disease. Currently available laboratory tests are not sufficient to justify nutritional intervention for patients with HCC. However, sarcopenia by balancing performance and nutritional status may predict the prognosis in patients with malignancies [5,6]. The Asian Working Group for Sarcopenia 2019 consensus defined sarcopenia as “loss of muscle mass, low muscle strength, and/or low physical performance” [19]. There are diagnostic tools useful in the assessment of muscle mass and sarcopenia in clinical and research settings [7,10]. One widely accepted method determines SMI from CT image at L3 [20]. Unfortunately, SMI evaluation requires dedicated software and can be time-consuming.

Recently, several studies reported single muscle, rather than total skeletal muscle, mass for sarcopenia assessment [11,21]. The most commonly used individual muscle for sarcopenia diagnosis is the psoas muscle. Parameters of the psoas muscle, such as density, area and thickness, while possibly useful in predicting prognosis, had not been assessed in relation to HCC patients treated with TACE. In the present study, PMI was calculated as the sum of the length and width of the psoas muscle, normalised by height squared. Either PMI or SMI proved to be similarly predictive of OS in the multivariate analysis (C-index 0.78, 0.79 for PMI and SMI, *p* = .985). Moreover, PMI has advantages compared to SMI being faster and easier to acquire, thus making it more likely to achieve widespread clinical application.

We propose that PMI can serve as a surrogate for the assessment of sarcopenia. The psoas muscle is the main flexor of the hip and also provides postural support of the lumbar spine, sacroiliac, and hip joints. Although it only accounts for approximately 10% of total skeletal muscle area at L3, the psoas is a key muscle for posture and core strength [22] and may more reliably reflect sarcopenia better than other individual muscle groups [23]. In addition, measuring psoas muscle may avoid the confounding effects while ascites accompanied [24].

Sarcopenia is a multifactorial condition related to age, reduced physical activity, and protein-energy malnutrition, but also to a severe wasting status due to malignant tumour or chronic disease [8,25]. In healthy adults, muscle loss is usually an ageing-related condition [8]. However, the progression of muscle loss is more severely accelerated once the tumour developed [25]. In the present study, PMI was significantly correlated with advanced tumour stage rather than age, suggesting that sarcopenia is not only a primary sign of ageing, but also a status of advancing disease. In addition, muscle reduction was more strongly associated with a poor prognosis than older age in HCC patients in our study. Typically, for HCC patients, neither inadequate intake and uptake kept the metabolic normality in balance, nor cancer-related inactivity result in the onset and progression of sarcopenia [5]. Mechanistically, sarcopenia develops as a result of protein energy malnutrition, a series of regulation among protein synthesis and breakdown, myostatin, reactive oxygen species and inflammatory cytokines [25]. The consequences in practice, generally, sarcopenia represents the result of a serious disruption of muscle metabolic balance due to decreased synthesis in liver cirrhosis and increased consumption of advanced tumours. It is also worth investigating these mechanisms for sarcopenia and their complex interactions deeply.

In the current study, AFP level, Child-Pugh class, maximum tumour diameter, metastasis, and BCLC stage were demonstrated to predict OS in HCC patients treated with TACE, which were similar to previously reported data [26,27]. However, aetiology and PVTT were not independent prognostic factor affecting the OS of HCC patients in the present study. Given these results in this study, we think there are several explanations. First, it has been reported that aetiology was a significant risk factor for the OS of patients with HCC [28]. However, the patients had heterogenous aetiologies, as the majority (85.1%) aetiology was HBV infection in the current study. Limited sample size in non-HBV populations possibly affected the results of multivariate analysis. Thus, aetiology is not an independent prognostic factor for OS in the present study. Second, PVTT was considered to be an important poor prognostic factor of HCC patients after TACE [26]. And the prognosis of these patients also depends on the subtypes of PVTT. HCC patients with main PVTT have more worse survival than those with branched PVTT [29]. In this study, 40 patients (17.5%) had PVTT, and the main and branched PVTT were 2 and 38 cases, respectively. PVTT was significantly associated with poor prognosis in univariate analysis. However, it was excluded in multivariate analyses. This is probably explained by the fact that BCLC C stage was a composite index and held greater weight than PVTT; thereby, the predictive value of PVTT for OS weakened in multivariate analyses. Besides, subgroup analyses according to the type of PVTT were not performed duo to the limited sample size.

The current study has several limitations. First, it is a retrospective study and as such secondary to *post hoc* analysis will have unavoidable biases. As well, some patients were excluded if CT scans were not available, which may result in selection bias. Third, the manual determination of PMI is subject to measurement error. This is likely due to the asymmetry of the muscle making determination of the length and width challenging. However, our results showed an excellent interobserver agreement with an ICC of 0.96 for assessment of PMI, indicating that, at least in our hands, the technique was reliable.

In conclusion, a retrospective analysis of PMI found that the approach was straightforward and accurate in predicting the long-term survival of HCC patients treated with TACE.

## Supplementary Material

Supplemental Material

## Data Availability

The authors confirm that the data supporting the findings of this study are available within the article and its supplementary materials.

## Abbreviations

**Abbreviations**
AFP: α-Fetoprotein
BCLC: Barcelona Clinic Liver Cancer
C-index: Concordance index
CI: Confidence interval
CT: Computed tomography
ECOG: Eastern Cooperative Oncology Group
HCC: Hepatocellular carcinoma
ICC: intraclass correlation coefficient
OS: Overall survival
PMI: Psoas muscle index
PVTT: Portal vein thrombus
SMI: Skeletal muscle index
TACE: Transarterial chemoembolization.
