# Supplementary material for: Low Psoas-Muscle index is associated with decreased survival in hepatocellular carcinoma treated with transarterial chemoembolization
Source: Ann Med. 2022 May 31;54(1):1562–9. doi: 10.1080/07853890.2022.2081872 (PMC9176702; doi:10.1080/07853890.2022.2081872)
Supplement: Supplemental Material [file IANN_A_2081872_SM5738.zip › suppl_data/Supplementary Tables.docx]

**Low Psoas-Muscle Index is Associated with Decreased Survival in Hepatocellular Carcinoma Treated with Transarterial Chemoembolization**

**Supplementary Table 1. Patients characteristics classified by PMI**

| **Variables** | **High PMI (n = 123)** | **Low PMI (n = 105)** | ***P* value** |
| --- | --- | --- | --- |
| Age (years) | 58.8 ± 11.1 | 58.9 ± 11.0 | 0.978 |
| Gender (male/female) | 90/33 | 85/20 | 0.166 |
| BMI (≥ 24 kg/m^2^) | 67 | 37 | 0.004 |
| Etiology (HBV/others) | 105/18 | 89/16 | 0.898 |
| AFP (> 400 ng/mL) | 34 | 44 | 0.024 |
| Child-Pugh class (A/B) | 113/10 | 88/17 | 0.060 |
| ALBI grade (1/2/3) | 69/51/3 | 35/65/5 | 0.003 |
| Number of tumors (> 3) | 29 | 34 | 0.138 |
| Maximum tumor diameter (> 3 cm) | 63 | 81 | < 0.001 |
| Portal vein thrombus | 13 | 27 | 0.003 |
| Metastasis | 12 | 18 | 0.10 |
| BCLC stage (A/B/C) | 40/60/23 | 18/52/35 | 0.007 |

Note: BMI = body mass index; AFP = α-Fetoprotein; BCLC = Barcelona Clinic Liver Cancer; PMI = psoas muscle index

**Supplementary Table 2. Patients characteristics classified by SMI**

| **Variables** | **High SMI (n = 139)** | **Low SMI (n = 89)** | ***P* value** |
| --- | --- | --- | --- |
| Age (years) | 56.2 ± 10.0 | 63.0 ± 11.2 | < 0.001 |
| Gender (male/female) | 99/40 | 76/13 | 0.013 |
| BMI (≥ 24 kg/m^2^) | 78 | 26 | < 0.001 |
| Etiology (HBV/others) | 122/17 | 72/17 | 0.155 |
| AFP (> 400 ng/mL) | 47 | 31 | 0.874 |
| Child-Pugh class (A/B) | 125/14 | 76/13 | 0.301 |
| ALBI grade (1/2/3) | 64/71/4 | 40/45/4 | 0.810 |
| Number of tumors (> 3) | 34 | 29 | 0.181 |
| Maximum tumor diameter (> 3 cm) | 78 | 66 | 0.006 |
| Portal vein thrombus | 17 | 23 | 0.621 |
| Metastasis | 15 | 15 | 0.186 |
| BCLC stage (A/B/C) | 43/67/29 | 15/45/29 | 0.027 |

Note: BMI = body mass index; AFP = α-Fetoprotein; BCLC = Barcelona Clinic Liver Cancer; SMI = skeletal muscle index
